# Advances of Zebrafish in Neurodegenerative Disease: From Models to Drug Discovery

**DOI:** 10.3389/fphar.2021.713963

**Published:** 2021-07-14

**Authors:** Xiaobo Wang, Jin-Bao Zhang, Kai-Jie He, Fen Wang, Chun-Feng Liu

**Affiliations:** ^1^ Department of Neurology, The Second Affiliated Hospital of Soochow University, Suzhou, China; ^2^ Jiangsu Key Laboratory of Neuropsychiatric Diseases and Institute of Neuroscience, Soochow University, Suzhou, China; ^3^ Department of Neurology, Suqian First Hospital, Suqian, China

**Keywords:** zebrafish, neurodegenerative disease, animal model, drug discovery, alzheimer’s disease, parkinson’s disease, amyotrophic lateral sclerosis

## Abstract

Neurodegenerative disease (NDD), including Alzheimer’s disease, Parkinson’s disease, and amyotrophic lateral sclerosis, are characterized by the progressive loss of neurons which leads to the decline of motor and/or cognitive function. Currently, the prevalence of NDD is rapidly increasing in the aging population. However, valid drugs or treatment for NDD are still lacking. The clinical heterogeneity and complex pathogenesis of NDD pose a great challenge for the development of disease-modifying therapies. Numerous animal models have been generated to mimic the pathological conditions of these diseases for drug discovery. Among them, zebrafish (*Danio rerio*) models are progressively emerging and becoming a powerful tool for in vivo study of NDD. Extensive use of zebrafish in pharmacology research or drug screening is due to the high conserved evolution and 87% homology to humans. In this review, we summarize the zebrafish models used in NDD studies, and highlight the recent findings on pharmacological targets for NDD treatment. As high-throughput platforms in zebrafish research have rapidly developed in recent years, we also discuss the application prospects of these new technologies in future NDD research.

## Introduction

Neurodegenerative disease (NDD), including Alzheimer’s disease (AD), Parkinson’s disease (PD), and amyotrophic lateral sclerosis (ALS), are progressive disorders with abnormal protein deposition and selective neuronal loss, leading to motor, and/or cognitive impairments and inevitable death (Vaquer-Alicea and Diamond, 2019). NDD has afflicted millions of people worldwide, and is one of the main reasons for the high incidence of mortality in elderly people (Hou et al., 2019). The World Health Organization (WHO) predicts that NDD will become the second leading cause of death after cardiovascular disease by 2040 (Gammon, 2014). Despite huge basic and clinical research efforts, no disease-modifying interventions are available to patients and therapeutic strategies are limited to symptomatic treatments at present. The main challenges in the study of NDD drug development are the diversity of etiology and clinical heterogeneity among individuals. In addition, the lack of biomarkers makes it difficult to identify the disease in the early stages. There is an increasing need for more animal models that can outline the complexity of NND pathogenesis.

Zebrafish (*Danio rerio*) were initially used to monitor water contamination, teratogenesis and toxic substances. In 1981, Streisinger *et al.* introduced zebrafish as an effective animal model especially in genetics and developmental biology (Streisinger et al., 1981). Thereafter, zebrafish were widely used by scientists in different fields of research (Lieschke and Currie, 2007). Zebrafish have several experimental advantages. First, their small body size and ease of breeding and maintenance make zebrafish ideal for high-throughput screening for genetic phenotypes and drugs. Second, their rapid development cycle and large spawning numbers greatly reduce time and cost, and provide sufficient samples to minimize differences between individuals. Third, transparent zebrafish embryos and larvae are particularly good for optical manipulation and imaging of neural activity. Finally, high genome homology to humans make zebrafish a valuable model for studying human disease in pharmacology, developmental genetics or other fields. The zebrafish is a perfect model in pharmaceutical science and is sensitive to many pharmacological agents.

Although zebrafish are different to mammals in organization structure of the central nervous system, several cerebral nuclei in the zebrafish brain including the basal ganglia, striatum, hippocampus and amygdala have high homogeneity to mammals (Randlett et al., 2015). The laminated cortex is not developed in zebrafish telencephalon, but the existence of the foxg1 gene which encodes telencephalon transcription factor indicates the high similarity in brain development between zebrafish and human. Neuroendocrine function in zebrafish hypothalamus is very similar to mammals and the zebrafish cerebellum also has a similar layer structure to that in humans. Moreover, the primary neurotransmitter system in zebrafish, including the noradrenergic, serotonergic, dopaminergic and histaminergic systems, show many similarities to the mammalian system (Pitchai et al., 2019). For example, D1 and D3 receptors in the dopamine (DA) system display complete amino acid homology in the binding site between zebrafish and humans, while D2 and D4 receptors show 85–95% homology. Therefore, it is helpful to establish zebrafish models to study the underlying mechanism of neuropsychiatric diseases and develop new medicine for treatment.

**FIGURE 1:**
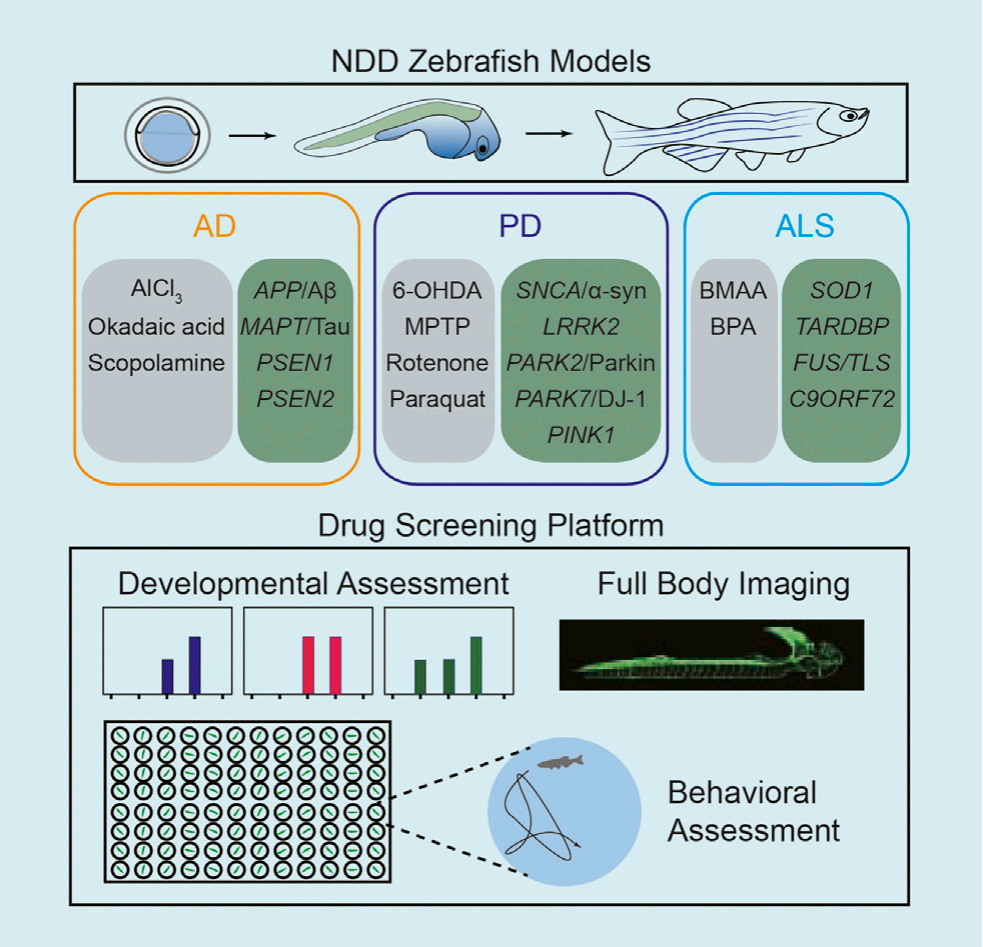
Schematic diagram of the application of zebrafish for high-throughput drug screening in NDD research.

In NDD, especially AD, PD, and ALS, zebrafish have been validated as a feasible tool for research use. Advances in novel imaging strategies, tractable behavioral tests and high-throughput drug screening methods in zebrafish, and more new drug targets have recently been discovered for the treatment of NDD. Zebrafish provide a useful platform for drug discovery in NDD that is time- and cost-efficient (Figure 1). In this review, we discuss the application of zebrafish as models in the pharmacology of AD, PD, and ALS.

## Behavioral Assessment of Zebrafish in Neurodegenerative Disease

Visible zebrafish behaviors are pivotal indicators of disease model establishment or drug efficacy verification at the organism level. Zebrafish embryos can quickly develop into larvae 72 h post fertilization (hpf). During this process, the complete body plan is established 48 hpf. Zebrafish undergo metamorphosis and are juvenile at 30 days post fertilization (dpf) and the adult phase starts from 90 dpf (Basnet et al., 2019). The brain-blood barrier (BBB) develops from 72 hpf onwards. Furthermore, zebrafish are active in the daytime and have physiological advantages in visual, auditory and olfactory function. Zebrafish and their larvae show different advantages or application in research. For example, large-scale high-throughput behavioral analysis has been carried out in larvae but not adult zebrafish, while adult zebrafish harbor a more sophisticated behavior repertoire than larvae (Basnet et al., 2019). Drug treatment of zebrafish is easy and convenient in the aqueous environment. The efficacy, bioavailability and toxicity of drugs can be evaluated in various steps (Vaz et al., 2018). However, the inadequacy of this model is that this drug administration route is different to humans and mammals where drugs are taken orally. The possibility of drug absorption through the skin or other organs can not be eliminated. This could lead to discrepancies in drug efficacy evaluation. A plethora of zebrafish behaviors in larvae and adult fish highlight zebrafish as a promising model for neurobiological investigations.

### Locomotor Activity

To measure the spontaneous swimming behavior of zebrafish, individuals are placed on a light and optical stage with an infrared light source. Digital videos are recorded to track the movement of zebrafish in water horizontally or vertically during a set time session. The swimming pattern of zebrafish can be observed by an automatically drawn trajectory in specialized software systems. Representative swimming behavioral parameters, such as mean velocity, total moving distance, or turning angle are analyzed simultaneously (Wang et al., 2017; Naini et al., 2018). A rough method of measuring zebrafish movement is counting the total number of lines that zebrafish cross. Vertical lines are drawn at equal distances to divide the vessel into several zones. This method is more suitable for adult zebrafish due to their less random behavior compared with larvae (Bretaud et al., 2004).

### Touch-Evoked Escape Response

Zebrafish larvae have a propensity to respond to visual, tactile, or acoustic stimuli by suddenly increasing the velocity and acceleration of movement. The sensorimotor reflexes are induced by touching the head, tail or pectoral fin of the larvae several times. This experiment is usually executed by gently touching the larvae with a fine needle or other blunt tipped equipment (Paquet et al., 2009; Cosacak et al., 2017; Pinho et al., 2019). Immediate swimming behavior after touch is recorded until the tail returns to the resting position. The escape response generally reflects the functional outcome of motor neurons of zebrafish larvae.

### Light-Dark Test

Movement pattern affected by alternating light and dark conditions reflects an anxiety-like behavior in zebrafish. Light to dark transition increases zebrafish locomotion while dark to light transition decreases locomotion. Zebrafish larvae can be placed in a 96-well plate with one larva per well for high-throughput drug screening. A chamber with a digital control can change the light and dark periods in cycles and monitor the movement of zebrafish larvae (Pan et al., 2019; Barbereau et al., 2020). Another method in larger adult zebrafish is to assess the time spend in the light compartment or the latency to enter the dark compartment. This test is conducted in a tank separated into equal sized light and dark areas with a plastic barrier. Zebrafish can swim freely between these two areas with barrier lift-up. Each zebrafish is initially placed in the light area, and the number of entries and time spent in the light area are assessed (Rishitha and Muthuraman, 2018; Lv et al., 2019).

### T/Y-Maze Test

Spatial discrimination such as in the T/Y-maze, has been used to characterize learning and memory in zebrafish similar to rodents. The T/Y-maze test utilizes positive stimulus as a reward to initiate motivation of zebrafish if they show the correct response in finding an objective. For example, the T-maze is composed of a starting zone, a long arm, two short arms and two chambers (Pu et al., 2017). Food is employed as a positive stimulus and delivered in one chamber for zebrafish training. The fish are starved for one or two days and then placed in the start zone to conduct the test. The behavior is recorded by camera and the time spent on finding the objective is quantified. A simple apparatus and procedure have been developed based on the T-maze (Williams et al., 2002; Koehler et al., 2018: Koehler et al., 2019). The tank is separated by a central white divider and adequate space at the bottom is reserved for zebrafish to swim freely between the two sections. A red card attached to one end allows fish to distinguish the two halves of the tank. The food is provided 5 s after a light tap at the center of the tank. Zebrafish present on the side with food within 5 s is considered the correct response.

### Novel Tank Diving Test

To assess the cognition of zebrafish, locomotor and exploratory activities are observed in the novel tank test. Zebrafish are individually placed in the novel tank and their swimming trajectory is recorded. The tank is divided into three horizontal sections which are defined as the bottom, middle and top areas, respectively. Vertical exploration by zebrafish can be measured by the number of entries and time spent in the bottom or top area. Moreover, anxiety-related behaviors can also be determined by the number and duration of freezing bouts or erratic movement in this test (Nunes et al., 2017; Zanandrea et al., 2018). Cuvettes can be used as novel apparatus which are placed in a parallel arrangement in front of the camera for large sample measurement (Feng et al., 2014).

## Alzheimer’s Disease

AD was first reported in 1907 and more than 95% of AD cases occur sporadically in adults who are over 65 years old (Tecalco-Cruz et al., 2020). The AD cases caused by genetic mutations only account for less than 1% while the majority of cases are related to environmental, genetic susceptibility and other factors. The main clinical characters of AD are memory loss, cognitive dysfunction and related comorbidities such as depression or anxiety. Cardinal pathological changes in AD are the death of neurons in brain regions responsible for cognitive functions, the presence of extracellular β-amyloid (Aβ) plaques and intracellular neurofibrillary tangles (NFTs). Based on previous findings, scientists have proposed several hot hypotheses that may induce the development of AD: the amyloid cascade hypothesis, tau hyperphosphorylation, the cholinergic hypothesis, neuroinflammation, and metal ions. Numerous pharmacological and transgenic (Tg) AD models have been established in zebrafish to investigate AD pathogenesis.

### Neurotoxic Zebrafish Models of Alzheimer’s Disease

Using environmental neurotoxins that trigger AD-like progression is a good approach for investigating AD etiology. Cholinergic dysfunction and cholinergic neuronal loss are observed in the brain of AD patients. Therefore, one hypothesis of AD occurrence is a decline in the synthesis of the neurotransmitter acetylcholine. Scopolamine, an acetylcholine muscarinic receptor antagonist, has been used to establish the zebrafish neurotoxin-induced AD model. Scopolamine can cause learning deficits in zebrafish and the effect can be reversed by acetylcholinesterase inhibitors, such as physostigmine (Kim et al., 2010). Acetylcholinesterase (AChE) and butyrylcholinesterase (BuChE) are enzymes which degrade acetylcholine and are involved in regulation of the cholinergic system. Both enzymes have been proved to contribute to AD progression (Hu et al., 2019).

Although the glutamatergic system and GABAergic system are associated with AD, the underlying mechanisms remain unclear. Glutamatergic neuronal loss in the medial temporal lobe and hippocampal network in AD shows that excitotoxicity of the glutamatergic system is associated with the disease (Greenamyre and Young, 1989). The effects of GABAergic compounds in ameliorating cognitive impairment induced by Aβ demonstrate the contribution of the GABAergic system to AD pathogenesis (Louzada et al., 2004).

Okadaic acid (OKA) is an inhibitor of protein phosphatase 2A (PP2A), which dephosphorylates tau and is associated with cognitive decline. In the AD zebrafish model induced by OKA, Aβ protein aggregation, senile plaque, increased p-tau and kinase glycogen synthase 3β (GSK-3β) are detected in zebrafish brain (Nada et al., 2016). Learning and memory deficits are also observed accompanied by these histological and pathological changes. This model represents most of the neuropathology related to AD and is easy to develop for exploring the mechanisms of AD and drug discovery.

Metal ions such as aluminum are thought to be neurotoxin and induce AD morbidity (Wang et al., 2016; Wang et al., 2020a). Zebrafish exposed to aluminum trichloride (AlCl_3_) show increased AChE activity at concentrations ranging from 50 to 250 μM and locomotor activity deficiency in acid conditions of pH 5.8 (Senger et al., 2011). This indicates that aluminum-induced AD is due to increased AChE activity. A recent study assessed the neurotoxic role of lead (Pb), a heavy metal, in zebrafish fibroblast cells (Lee and Freeman, 2020). The findings suggested that Pb exposure results in *de novo* DNA copy number alterations in a dose-dependent manner. Although most of the genes in the copy number alterations regions were related to the amyloid precursor protein (APP) molecular pathway, whether Pb is involved in neurological disease needs further elucidation, particularly in AD.

### Genetic Zebrafish Models of Alzheimer’s Disease

Aβ is generated from APP which is a type I transmembrane glycoprotein. β- and γ-secretases participate in the cascade cleavage of APPβ to form Aβ peptides, whereas α- and γ-secretases are involved in P3 peptide production from APPα. Various isoforms of Aβ with different lengths are produced, especially the toxic peptides Aβ42 and Aβ40. Oligomers, protofibrils, and fibrils generated by the accumulation of Aβ finally lead to Aβ plaque formation. Cerebroventricular microinjection of Aβ42 into zebrafish brain induces Aβ protein accumulation, apoptotic cell death, microglia activation and synaptic degeneration (Bhattarai et al., 2017). Islet amyloid polypeptide (IAPP) or Aβ microinjection into zebrafish embryos can produce an AD-like animal model to study amyloidosis (Nery et al., 2014). This method provides an acute AD model for cost-effective and efficient neuropathology study and drug screening in the future. The Swedish mutant *APP* is known to cause familial AD and zebrafish harbor two genes (*appa* and *appb*) that are similar to human *APP* (Musa et al., 2001). Although *APP* gene is evolutionary conserved in vertebrates, the function of *appb* is more paramount than *appa* since loss of *appb* not only impair locomotor behavior, motor neuron patterning and formation, Mauthner cell development but also disrupt cell adhesion during early development in zebrafish (Abramsson et al., 2013; Banote et al., 2016; Banote et a2020). Tg Swedish mutant *APP* zebrafish with *appb* promoter successfully express the disease-causing protein in brain, heart, eyes and vasculature. This model exhibits AD-like behavioral symptoms and cerebral β-amyloidosis, as well as neuronal loss and an enlarged perivascular space (Pu et al., 2017). Clearly elucidating the feature of zebrafish gene duplication would pave the road for mechanism exploration of key disease related genes which are potentially lethal in mammals.

NTFs are another typical hallmark of AD and are composed of highly phosphorylated tau. Tau is a microtubule-associated protein which is involved in modulating microtubule assembly and stabilization (Uddin et al., 2020). Hyperphosphorylated tau protein tends to form NTFs and affects microtubule normal function, microtubule dynamics and axonal transport (Sonawane and Chinnathambi, 2018). A stable Tg zebrafish model with mutations on tau encoding gene *MAPT* (microtubule-associated protein tau), such as Tau-P301L and Tau-A152T, has been established to mimic AD key pathological features of tauopathies. The mutations are found in AD patients with frontotemporal dementia. Tg zebrafish models under the control of a neuronal-specific HuC promoter is used to study AD pathogenesis and drug discovery. Gal4/UAS-based human Tau-P301L-Tg zebrafish show tau hyperphosphorylation, aggregation, degeneration, and behavioral deficiency (Paquet et al., 2009). In Gal4/UAS-based human Tau-A152T-Tg zebrafish, higher tau phosphorylation, neurodegeneration and impaired proteasome activity have been detected. Autophagy plays a role in tau clearance in this Tg model (Lopez et al., 2017). However, in Cre/LoxP-based Tau-P301L-Tg adult zebrafish, no oligomers, NTFs or symptoms of tauopathies were observed only tau hyperphosphorylation (Cosacak et al., 2017). It is possible that different Tg systems or development stages of zebrafish influence the model traits. In adult human brain, six isoforms of tau due to alternative splicing have been identified. Lack of microtubule-binding repeat, encoded by exon 10, leads to the generation of three isoforms with three repeats (3R), while its inclusion produces another three isoforms with four repeats (4R). An additional 29 or 58 amino acids encoded by exons 2, or exons 2 and 3 distinguish three isoforms with the same repeats (Goedert and Spillantini, 2019). In zebrafish, two paralogues by duplication of an ancestral teleost *MAPT* ortholog, *mapta* encodes tau isoforms with four, five or six repeats and *maptb* mainly encodes isoforms with 3R. *Mapta* and *maptb* display different expression patterns in certain brain regions during develoment, suggesting a subfunctionalization role of *MAPT* orthologue after duplication. For instance, *mapta* mRNA is present in ganglion neurons of the retina while *maptb* is not (Chen et al., 2009). Wu *et al.* attempted to overexpress zebrafish 3R or human 4R in zebrafish to observe tauopathy pathogenicity. They found both wild-type and truncated forms are prone to aggregate and increase neuronal death (Wu et al., 2016). More efforts are needed to develop AD-like models by modifying the expression of different tau isoforms. Protein presenilins (PSEN) 1 and 2 are highly homologous proteins, that act as potential catalytic subunits of γ-secretase (Walter et al., 1998). Mutations in their coding genes *PSEN1* and *PSEN2*, have been identified in early-onset familial AD patients. Mutant *PSEN1* or *PSEN2* have been identified to increase longer and highly fibrillogenic Aβ formation in the brain to induce the onset of AD (Kabir et al., 2020). The zebrafish orthologs of *PSEN1* and *PSEN2* are *psen1* and *psen2.* Deletion of *psen1* in zebrafish causes histamine neuronal loss, and the histaminergic system is suggested to mediate cognitive functions in AD (Sundvik et al., 2013). *psen1* splicing interference in zebrafish can induce early-onset AD phenotypes, such as cognitive deficit, Aβ42 aggregation and synaptic reduction (Nery et al., 2017). Blocking *psen2* translation, results in defects of zebrafish embryo development in the central nervous system with Notch signaling interference (Nornes et al., 2009). Moreover, the zebrafish genome edited to model heterozygous K115fs mutation of human *PSEN2* exhibits accelerated brain aging and microglia-associated immune responses without obvious histopathology (Hin et al., 2020). Further solid evidence of *PSEN1* and *PSEN2* function in zebrafish is mandatory to allow the application of this Tg model in drug discovery.

### Drug Discovery in Zebrafish Models of Alzheimer’s Disease

GSK3 is known to phosphorylate tau on multiple sites and its isoform GSK-3β is crucial for abnormal tau hyperphosphorylation. GSK3β in zebrafish is functionally similar to human GSK3β. The GSK-3β inhibitor, lithium chloride can reverse cognitive deficits and tau hyperphosphorylation in an *in vivo* AD model, which was established by injecting Aβ42 into the hindbrain ventricle of zebrafish embryos (Nery et al., 2014). Lithium chloride is also used as a positive control to protect zebrafish locomotors or dephosphorylation for tauopathy in the Tau-P301L-Tg zebrafish model (Naini et al., 2018; Barbereau et al., 2020). Another form of lithium, lithium carbonate (Li_2_CO_3_), has been demonstrated to prevent memory impairment and AChE activity in zebrafish induced by scopolamine (Zanandrea et al., 2018). The commercially available compound, TDZD-8 can reduce zebrafish mortality rate and improve cognitive impairment induced by OKA. Protein levels of p-tau, PP2A and the ratio of active: inactive GSK3β are also reduced following treatment with TDZD-8 (Koehler et al., 2019). The synthesized GSK3 inhibitor AR-534 can reduce tau hyperphosphorylation in the Tau-P301L mutation zebrafish model (Paquet et al., 2009). In discovering new GSK inhibitors, AR-A014418, compounds 9e and 26 days, Monte *et al.* used zebrafish embryos to evaluate the safety and permeability of these inhibitors (Monte et al., 2011; Lo Monte et al., 2013). The results corroborate the use of GSKβ inhibitors in the treatment of AD. However, there is a lack of evidence to decide which GSKβ inhibitor is most efficacious. Notably, GSK3 inhibitor treatment results in zebrafish embryos lacking eyes through activation of the Wnt signaling pathway (Atilla-Gokcumen et al., 2006; Zhong et al., 2009).

Regulation of the cholinergic system can suppress cholinesterase activity and provide guidance in AD treatment. A number of drugs have been identified and investigated in terms of this mechanism. Cotinine and 6-hydroxy-l-nicotine were found to attenuate zebrafish anxiety-like behavior and memory impairment in a scopolamine-induced model. Oxidative stress and AChE activity were also reduced in the brain of zebrafish (Boiangiu et al., 2021). In the same zebrafish model with memory impairment, *Thymus vulgaris* L. essential oil (TEO) ameliorated zebrafish amnesia and anxiety, reduced AChE activity and brain antioxidant capacity. The compounds contained in TEO are mainly thymol and *p*-cymene as shown by gas chromatography analysis (Capatina et al., 2020). Linarin, an active ingredient of the flavonoid glycoside, can protect against AlCl_3_-induced zebrafish dyskinesia by inhibiting AChE activity (Pan et al., 2019). Molecular docking simulation showed that linarin can bind with AChE active sites. Compounds designed from 3-(4-aminophenyl)-coumarin, especially compound 4m, alleviated behavioral symptoms and blocked AChE and BuChE activity (Hu et al., 2019). Compound 4m is suggested to be an interesting lead for drug design in AD treatment.

Tau hyperphosphorylation prevention allows the discovery of novel anti-tauopathy drugs against AD. A cyclin-dependent kinase 5 (CDK5) inhibitor, LDC8, can protect against neurodegeneration, neuroinflammation and synapse density in the Aβ42-induced AD zebrafish model by modulating the phosphorylation of microtubule-associated proteins (Reinhardt et al., 2019). Heparan sulfate is a highly sulfated polysaccharide which is associated with tau aggregates in AD (Naini and Soussi-Yanicostas, 2018). Treatment with surfen or its derivative oxalyl surfen, both possess heparan sulfate antagonist properties, can decrease tau hyperphosphorylation and rescue neuronal and behavioral deficits in Tg [HuC:hTauP301L; DsRed] Tg zebrafish (Naini et al., 2018). Decreased tau hyperphosphorylation observed with certain GSK-3β inhibitors suggests p-tau inhibition which is probably a common way of alleviating AD (Paquet et al., 2009; Nery et al., 2014; Zanandrea et al., 2018; Koehler et al., 2019).

A number of drugs are promising as they show multi-effects in AD-associated symptoms and neuropathology attenuation. 3-arylcoumarin is a compound which has a coumarin skeleton and an aryl structure at the 3-position. 3-arylcoumarin derivatives show dual cholinesterase and monoamine oxidase (MAO) inhibition and improved juvenile zebrafish movement in the AlCl_3_-induced zebrafish model (Yang et al., 2019). Ferulic acid is an antioxidant which possesses a variety of functions, including amyloid structure elimination. Dyskinesia and the reaction capacity of AlCl_3_-induced zebrafish recovered following administration of the synthesized ferulic acid derivative, TM-10. TM-10 shows influence on BuChE, MAO and self-induced Aβ aggregation. Moreover, memory loss is improved by TM-10 in scopolamine-induced zebrafish and vascular injury is protected in Aβ40-induced zebrafish (Sang et al., 2019). The newly designed and synthesized cinnamic acid hybrid, compound 4e, can inhibit Aβ42 accumulation, and shows reversible BuChE activity and MAO-B inhibitory activity. Compound 4e improves dyskinesia recovery rate and reaction capacity in AlCl_3_-induced AD zebrafish models (Wang et al., 2021). A natural sulfur amino acid metabolite, LKE, rescued the cognitive defects and cell apoptosis in the dorsal lateral pallium in the OKA-induced AD zebrafish model (Koehler et al., 2018). In addition, LKE increased BDNF expression, phosphorylation of PKB/Akt and cAMP response element binding protein (CREB).

Studies have also been conducted to develop nanomedicine against AD or human amyloid diseases and the zebrafish model has been used for economic and high-throughput drug screening. In the Aβ toxicity induced zebrafish larvae model, casein coated-gold nanoparticles (βCas AuNPs) were designed and tested (Javed et al., 2019). βCas AuNPs can sequester intracerebral Aβ42 to mitigate Aβ toxicity in a nonspecific and chaperone-like manner. Disappearance of amyloid plaque formation, reduced reactive oxygen species (ROS) and the rescue of synaptophysin loss were observed. Moreover, βCas AuNPs rescued the mobility and cognitive function in the adult zebrafish model of Aβ toxicity. Carbon quantum dots (CQDs) are zero-dimensional, carbon-based nanostructures for carrying drugs (Koppel et al., 2020). CQDs can rescue impaired zebrafish embryonic hatching and suppress the production of ROS in the organism. Carbon dots in this study function as an Aβ inhibitor by deactivating β-secretase 1 (BACE1) (Han et al., 2017).

Although the γ-secretase inhibitor (GSI), DAPT, was developed to prevent Aβ production via interference with PSEN function, potential toxic side effects were observed (Nornes et al., 2009). GSIs not only inhibit Aβ generation in zebrafish embryos, but also impact the Notch signaling pathway which plays a paramount role during embryonic development and throughout adulthood. However, there are also GSIs, such as the JLK isocoumarin inhibitors, which aim to reduce Aβ production without interfering with the Notch signaling pathway (Petit et al., 2003; Arslanova et al., 2010; Yang et al., 2010). Therefore, AD-related drugs should be designed to minimize side effects without triggering an unwanted pathway.

All the compounds described above are summarized in Table 1.

**TABLE 1:** Drugs identified for AD treatment in zebrafish models.

Compound	Classification	Model	Effects on phenotype	Behavioral method	Molecular mechanism	References
Surfen and oxalyl surfen	Heparan sulfate antagonist	Tau-P301L-Tg	Rescue impaired escape response; Rescue motor neuron defects	Touch-evoked escape response, 48 hpf	Decrease p-tau	Naini et al. (2018)
AR-534	GSK-3β inhibitor	Tau-P301L-Tg	Not applicable	Touch-evoked escape response, 48 hpf	Decrease p-tau	Paquet et al. (2009)
Linarin	Flavonoid glycoside	AlCl_3_	Protect against dyskinesia	Light-dark test, 3 dpf	Inhibit AChE activity	Pan et al. (2019)
TDZD-8	GSK-3β inhibitor	OKA	Reduce mortality rate; Reverse cognitive deficits	Spatial discrimination test, 12–15 months	Decrease p-tau, PP2A and the ratio of active: Inactive GSK3β	Koehler et al. (2019)
LKE	Lanthionine ketimine derivatives	OKA	Reverse cognitive defects; Resist cell apoptosis	Spatial discrimination test, 12–15 months	Increase BDNF, pPKB/Akt and pCREB	Koehler et al. (2018)
Li_2_CO_3_	GSK-3β inhibitor	Scopolamine	Reverse cognitive deficits	Novel tank test, fish; Y-maze task, 6 months	Decrease p-tau	Zanandrea et al. (2018)
Compound 4m	3-Arylcoumarin derivatives	AlCl_3_	Alleviated behavior symptoms	Locomotion test with trajectory recording; 72 hpf	Inhibit AChE and BuChE activity	Hu et al. (2019)
LiCl	GSK-3β inhibitor	Aβ42 injection; Tau-P301L-Tg	Reverse cognitive deficits	Avoidance learning; touch-evoked escape response, 5 dpf	Decrease p-tau	Nery et al. (2014)
Cotinine/6-hydroxy-l-nicotine	Nicotine derivate	Scopolamine	Rescue anxiety-like behavior and memory impairment	Novel tank test, 15–16 days; Y-maze task, 17–18 days; Object discrimination test, 19–23 days	Reduce oxidative stress and AChE activity; upregulate npy, egr1, bdnf, nrf2a mRNA levels	Boiangiu et al. (2021)
TEO	*Thymus vulgaris* L	Scopolamine	Reduce amnesia and anxiety	Novel tank test, 7 days; Y-maze task, 8 days; Object discrimination test, 9–13 days	Reduce AChE activity and brain antioxidant capacity	Capatina et al. (2020)
LDC8	CDK5 inhibitor	Aβ injection	Reduce neurodegeneration, neuroinflammation and protect synapses	Not applicable	Decrease p-tau; inhibit GSK3β and CDK5	Reinhardt et al. (2019)
TM-10	Ferulic acid derivatives	AlCl_3_; Aβ injection	Improve response efficiency and exercise capacity; prevent memory loss	Light-dark test, 3 dpf	Inhibit BuChE, MAO activity; Disaggregate Aβ	Sang et al. (2019)
Compound 4e	Cinnamic acid hybrids derivates	AlCl_3_	Improve dyskinesia recovery rate and reaction capacity	Locomotion test, 2 dpf	Inhibit Aβ42 accumulation, reverse BuChE activity, inhibit MAO-B activity	Wang et al. (2021)
βCas AuNPs	Nanoparticles	Aβ injection	Rescue motility and cognitive dysfunction	Swimming behavior with response to tap stimuli, 5 dpf	Eliminate amyloid plaque formation; reduce ROS; recover synaptophysin	Javed et al. (2019)
CQDs	Nanostructures	IAPP and Aβ microinjection	Rescue impaired zebrafish embryonic hatching	Not applicable	Suppress ROS production, deactivate BACE1	Han et al. (2017)

## Parkinson’s Disease

PD is a common neurodegenerative disease, affecting 2–3% of the population aged over 65 years (Hou et al., 2019). It is mainly characterized by motor deficits including bradykinesia, rigidity, resting tremor and abnormal gait. PD patients are also affected by non-motor symptoms such as hyposmia, depression, constipation and sleep disorder. The major pathological features of PD are neuronal loss in the substantia nigra pars compacta (SNpc) and the presence of Lewy bodies (LBs) consisting of misfolded α-synuclein (α-syn). The etiology of PD is still not known, but both genetic and environmental factors are thought to affect the progression of PD. At present, there is still no ideal treatment to delay the development of the disease. Dopamine precursor (l-DOPA) replacement is the most effective treatment for improving motor function; however, long-term application of the drug will worsen dyskinesia (Armstrong and Okun, 2020). PD is a heterogeneous disease with various pathophysiological changes in individuals. No model can accurately mimic all aspects of PD and different models possess their own unique advantages. Zebrafish have been utilized to investigate both neurotoxic and genetic effects in PD for more than 20 years and show great value in screening potential therapeutic drugs.

### Neurotoxic Zebrafish Models of Parkinson’s Disease

Neurotoxins induce rapid loss of nigrostriatal dopaminergic neurons in animal models. The most commonly used neurotoxins include 6-hydroxydopamine (6-OHDA), 1-methyl-4-phenyl-1,2,3,6-tetrahydropyridine (MPTP), rotenone, and paraquat. 6-OHDA is a hydroxylated compound of DA which is highly oxidizable and it can produce harmful ROS in the brain. Intracranial administration of 6-OHDA can successfully model motor disturbance and lead to a decrease in DA and noradrenaline levels in adult zebrafish (Anichtchik et al., 2004). Further studies have elucidated the neuronal oxidation and dopaminergic neuron loss in this model (Vijayanathan et al., 2017). Exposure to 6-OHDA also decreases tyrosine hydroxylase (TH) level and locomotor activity in zebrafish larvae or embryos (Parng et al., 2007; Feng et al., 2014). A recent study also revealed that 6-OHDA can induce ferroptosis in zebrafish brain (Sun et al., 2020).

MPTP was first discovered when addicted individuals with MPTP administration suddenly showed parkinsonian symptoms (Langston et al., 1983). It is oxidized into 1-methyl-4-phenylpyridinium ion (MPP^+^) by the MAO-B in astrocytes, and MPP^+^ is then quickly absorbed by dopaminergic neurons through the dopamine transporter and inhibits mitochondrial respiration complex I, leading to apoptosis and necrosis of DA neurons. Several studies have reported that MPTP administration kills DA neurons in embryonic, larval, and adult zebrafish (Anichtchik et al., 2004; Bretaud et al., 2004; Lam et al., 2005). Using MPTP-induced zebrafish models combined with Tg fluorescent labeling, studies have explored the topographic pattern of monoaminergic neuronal loss and the dynamics of mitochondrial transport after MPTP or MPP^+^ exposure (Wen et al., 2008; Xi et al., 2011; Noble et al., 2015; Dukes et al., 2016; Kalyn et al., 2019). These studies have deepened our understanding of the progression of PD and provided a good platform for the establishment of high-throughput drug screening in zebrafish.

Rotenone is a pesticide commonly used in farming and fishing. Researchers have found that chronic exposure to rotenone is associated with a higher risk of PD (Betarbet et al., 2000). Administration of rotenone in zebrafish can reduce DA neurons (Bretaud et al., 2004). Encouragingly, studies in recent years have found that the rotenone-based zebrafish model can mimic both motor and non-motor PD-like symptoms (Wang et al., 2017; Lv et al., 2019). This may provide a new approach to studying the pathology of early stage PD.

Paraquat, also a pesticide, has high capability to generate superoxide in mitochondria. Paraquat induces neurodegenerative phenotypes and the reduction of DA levels in zebrafish (Nellore and Nandita, 2015; Nunes et al., 2017), while the loss of DA neurons has not been reported in this model. Researchers have also developed mitochondria-targeted redox cycler paraquat (named MitoPQ) to selectively induce mitochondrial superoxide production, and model metabolic and neurodegenerative disease phenotypes (Robb et al., 2015). This toxin could be a useful tool for investigating novel drug targets for improving mitochondrial function (Pinho et al., 2019).

Zebrafish are also becoming a popular tool for evaluating the influence of environmental factors on PD pathogenesis. Exposure of zebrafish embryos to graphene-family nanomaterials has been reported to induce neurotoxicity and alter the expression of PD-related genes (Cao et al., 2021). Coincidentally, zinc oxide nanomaterials disrupt locomotor activity and induce apoptosis and neuronal loss in zebrafish (Jin et al., 2021). More recently, other researchers have also suggested that long-term exposure to a heterocyclic amine from a wide range of pollutants, contributes to the potential risk of PD (Li et al., 2021).

### Genetic Zebrafish Models of Parkinson’s Disease

Although neurotoxic models are feasible for defining potential symptomatic benefits for drug development, it is difficult to model the molecular pathology of PD. Family studies have discovered more than 20 risk genes associated with PD (Blauwendraat et al., 2020). In recent years, genome-wide association studies (GWAS) have also identified 90 independent risk variants which contribute to sporadic PD (Blauwendraat et al., 2020). The easiest approach used to study loss-of-function mutations in zebrafish is the injection of antisense morpholino oligonucleotides. Later, Tg and viral zebrafish models based on the genetic causes were widely used to study PD pathobiology and evaluate potential therapeutics.

*SNCA* (encoding α-syn, which is the major component of LBs) was the first gene found to be unequivocally linked to familial PD. Although zebrafish do not express α-syn ortholog, they express β-, γ1-, and γ2-synucleins and γ1-synuclein has a similar function to α-syn. Knockdown of β- and γ1-synucleins results in abnormal development of dopaminergic neurons and reduces DA levels, and expression of human α-syn in zebrafish can revert the phenotypes (Milanese et al., 2012). Combined with neurotoxic models, a recent study found that insufficient expression of human α-syn can amplify cytoplasmic peroxide flux and oxidative stress, causing motor abnormalities prior to neuronal loss (Kang et al., 2018; Van Laar et al., 2020). α-syn is still an appealing target for disease modification in PD. However, all existing zebrafish PD models can only produce a dopaminergic cell-loss phenotype and neither exhibits the formation of LBs.

Mutations in the *leucine-rich repeat kinase 2* (*LRRK2*) gene are implicated in both familial and sporadic cases of PD and the *LRRK2-G2019S* mutation is the most common (Blauwendraat et al., 2020). Zebrafish express a homolog of the human *LRRK2* gene, which contains all the functional domains. Disruption of LRRK2 in zebrafish leads to severe developmental defects and neuron loss (Prabhudesai et al., 2016). However, studies do not show consistent results on the effects of depleting the WD40 domain of LRRK2 (Sheng et al., 2010; Ren et al., 2011). To date, no zebrafish model has been generated to elucidate the effects of increased kinase activity of LRRK2 which is highly relevant to PD etiology.

Parkin is an E3 ubiquitination ligase encoded by *PARK2* gene. It mediates the ubiquitination process of many proteins including α-syn, and promotes the proteasome-dependent degradation and mitochondrial autophagy. Therefore, parkin plays an important role in the elimination of damaged proteins and misfolded proteins. Reduction of parkin activity in zebrafish leads to a significant decrease in the number of dopaminergic neurons, and parkin knockdown zebrafish embryos show specific impairment of the mitochondrial respiratory chain complex I (Flinn et al., 2009; Fett et al., 2010). Moreover, increased expression of parkin protected zebrafish from cell death (Fett et al., 2010).

DJ-1 (encoded by *PARK7*) appears to have multiple roles in protecting neurons from oxidative stress and loss-of-function of the protein can cause a rare form of early-onset PD. Knockdown of DJ-1 using antisense morpholino oligonucleotides in zebrafish embryos does not reduce the number of dopaminergic neurons, but leads to increased susceptibility of dopaminergic neurons to H_2_O_2_ (Bretaud et al., 2007). The loss of DJ-1 in the mouse does not reduce the number of dopaminergic neurons and locomotor activity; however, *PARK7* knockout zebrafish produce a reliable phenotype of PD (Edson et al., 2019; Hughes et al., 2020).

*PINK1* is another mitochondrial-related gene linked to early-onset PD. Like *PARK7*, knockdown of *PINK1* alone results in reduced dopaminergic neurons in zebrafish, and the models can successfully mimic PD pathology when incorporated with other PD-risk factors (Zhang et al., 2017; Brown et al., 2021). Flinn *et al.* also identified a fish line with a premature stop mutation (Y431*) in the Pink1 kinase domain, which induces mitochondrial dysfunction and loss of dopaminergic neurons (Flinn et al., 2013; Soman et al., 2017).

In addition, a variety of zebrafish Tg models have been generated based on the PD-associated genes recently. The loss-of-function of the *PARLA* gene decreases dopaminergic neurons mainly in the olfactory bulb (Merhi et al., 2021). VPS41 knockout causes lysosomal abnormalities as well as microglial and cerebellar dysfunction (Sanderson et al., 2021). *NUS1* knockdown zebrafish exhibit movement deficits caused by defective efflux of cholesterol from lysosomes (Yu et al., 2021). Zebrafish Tg models tend to be an effective tool to classify PD into different subtypes according to different pathogenic factors.

### Drug Discovery in Zebrafish Models of Parkinson’s Disease

By employing the above models, a multitude of drugs have been screened out and proved to be potentially effective in treating PD (Table 2). MAO-B is one of the enzymes that deaminate monoamine neurotransmitters like dopamine, noradrenaline, and serotonin in the brain. The age-related high expression of MAO-B is associated with mitochondrial dysfunction and reduction in the viability of DA neurons. Moreover, recent studies have also shown that α-syn can bind and activate MAO-B, accelerating PD progression. MAO-B inhibitors, such as l-deprenyl and rasagiline, show potential anti-dyskinetic and neuroprotective effects in PD zebrafish models (McKinley et al., 2005; Cronin and Grealy, 2017; Vaz et al., 2020). MAO-B inhibitors have been used clinically as an alternative to l-DOPA. Recently, more MAO-B inhibitors with high potency and selectivity have been designed to improve their therapeutic benefits and decrease the secondary effects. Studies in zebrafish models should be carried out to provide further evidence and accelerate the drug development process.

**TABLE 2:** Drugs identified for PD treatment in zebrafish models.

Compound	Classification	Model	Effects on phenotype	Behavioral method	Molecular mechanism	References
Trifluoperazine	Piperazine phenothiazine	Rotenone and PINK1-Tg	Protect dopaminergic neurons and mitochondrial function	Touch-evoked escape response, 2 dpf	Stimulate transcription factor EB nuclear translocation and enhance autophagy	Zhang et al. (2017)
Ruthenium red	MCU inhibitor	PINK1-Y431*-Tg	Prevent dopaminergic neuronal cell loss	Not applicable	Normalize mitochondrial function	Soman et al. (2017)
l-deprenyl	MAO-B inhibitor	MPTP	Protect dopaminergic neurons	Not applicable	Not applicable	McKinley et al. (2005)
Minocycline	Tetracycline analog	6-OHDA	Prevent locomotor deficits and neuronal loss	Light-dark test, 5 dpf	Not applicable	Cronin and Grealy, (2017)
Rasagiline	MAO-B inhibitor	6-OHDA	Prevent the locomotor deficits and neuronal loss	Light-dark test, 5 dpf	Not applicable	Cronin and Grealy, (2017)
Melatonin	Methoxy indole	MPTP	Improve motor activity	Locomotion test, 5 dpf	Restore the parkin/Pink1/DJ-1/MUL1 network	Díaz-Casado et al. (2016)
Rifampicin	Macrocyclic antibiotic	Rotenone	Improves neuroinflammation and mitochondrial function	Swimming behavior calculated by number of lines crossed, 6 months	Anti-inflammatory	Yurtsever et al. (2020)
LP17	Blocker of TREM-1	6-OHDA	Improve locomotion; partially protect dopaminergic neurons	Swimming behavior calculated by moving height, 5 dpf	Activate autophagy and anti-inflammatory	Feng et al. (2019)
CsAE	*C. siliqua*	6-OHDA	Improve cognitive function	Novel tank diving test, 7 days; Y-maze test, 8 days	Antioxidant and anti-AChE activities	Abidar et al. (2020)
Rosmarinic acid	Natural product	MPTP	Protect dopaminergic neurons and improve locomotor behavior	Locomotion test, 5 dpf	Attenuate the increases of ROS via regulation of the DJ-1/Akt/Nrf2 signaling	Zhao et al. (2020)
Fucoxanthin	Marine carotenoid	6-OHDA	Improve the locomotion	Locomotion test, 7 dpf	Decrease ROS levels via regulation of Keap1/Nrf2 signaling	Wu et al. (2021)
Naringenin	Flavanone	6-OHDA	Improve the locomotion	Locomotion test, 7 dpf	Regulate the expression of parkinsonian genes	Kesh et al. (2021a)
Theacrine	Purine alkaloid	MPTP	Retrieve dopaminergic neurons loss; improve locomotive activity	Touch-evoked escape response, 5 dpf	Activate SIRT3	Duan et al. (2020)
Hesperidin	Flavanone glycoside	6-OHDA	Improve locomotion	Locomotion test, 7 dpf	Downregulate lrrk2, gsk3β, casp3 and casp9	Kesh et al. (2021b)
EuO	Eucommia ulmoides Oliver	MPTP	Retrieve the loss of dopaminergic neurons and improve locomotion	Locomotion test, 7 dpf	Activate autophagy and contribute to α-syn degradation	Chen et al. (2020)
Hexane	*S. mombin*	Rotenone	Restore anxiety behavior	Novel tank test; light-dark test, 1 month	Prevent oxidative stress	Santo et al. (2021)

Mitochondria have an important role in energy metabolism and preserve redox homeostasis in response to stress. Excessive mitochondrial dysfunction is implicated in the pathology of PD. Combining Pink1 deficiency with rotenone, Zhang *et al.* developed a multifactorial zebrafish drug-screening method to compromise mitochondrial function and identified trifluoperazine and other piperazine phenothiazines as protective compounds (Zhang et al., 2017). Another study found that administration of melatonin can rescue zebrafish embryos from the MPTP-induced PD phenotype by restoring the parkin/Pink1/DJ-1/MUL1 function (Díaz-Casado et al., 2016). These studies will pave the way for future PD therapies that specifically target mitochondria.

Autophagy is a catabolic process that removes damaged organelles and aggregated proteins via the autophagy-lysosome pathway. Many studies have demonstrated that α-syn aggregates are mainly degraded via autophagy. In zebrafish, down-regulation of autophagy-related gene 5 (ATG5) results in the upgrade of PD-associated proteins, abnormal locomotor behavior and neuronal loss, while ATG5 overexpression alleviated these PD pathological features (Hu et al., 2017). This study revealed that up-regulation of autophagy-related genes may be an effective way of preventing PD progression. For instance, trifluoperazine can slow neurodegeneration via enhancing autophagy in response to mitochondrial stress (Zhang et al., 2017).

An imbalance of calcium homeostasis plays a vital role in PD pathogenesis. The vulnerability of dopaminergic cells in PD is related to overloaded cytosolic and mitochondrial calcium. A study reported that pharmacological inactivation of the mitochondrial calcium uniporter (MCU) by Ruthenium red, prevents neuronal loss in PINK1-Y431* mutant zebrafish (Soman et al., 2017). However, isradipine, a calcium channel blocker, did not reverse 6-OHDA-induced phenotypes in a zebrafish model (Cronin and Grealy, 2017). These studies assume that the effects of calcium channels may vary in PD with different etiologies. Owing to the importance of calcium in PD pathology, we suggest that future studies should be performed to verify the effects of more types of calcium channels, including synaptic receptors, in zebrafish models.

Although the relationship between neuroinflammation and PD is unclear, a growing body of evidence supports the immune-targeted therapies as promising PD treatments. An epidemiological study has confirmed the beneficial role of non-steroidal anti-inflammatory drugs in PD. Many researchers use zebrafish models to verify whether anti-inflammatory drugs are effective in improving the motor symptoms of PD. Minocycline, a tetracycline analog prevents the locomotor deficits and neuronal loss in the 6-OHDA-induced zebrafish model (Cronin and Grealy, 2017). Recent studies have reported that rifampicin, a macrocyclic antibiotic, improves neuroinflammation and mitochondrial function (Yurtsever et al., 2020) and LP17, a synthetic peptide blocker of triggering receptors expressed on myeloid cells 1 (TREM-1), shows neuroprotective effects via activation of autophagy and anti-inflammatory activity (Feng et al., 2019).

In addition, numerous compounds extracted from natural products have been screened out and verified to be effective in ameliorating PD pathology using zebrafish models (Abidar et al., 2020; Chen et al., 2020; Duan et al., 2020; Xiong et al., 2020; Zhang et al., 2020; Zhao et al., 2020; Kesh et al., 2021a; Kesh et al., 2021b; Kozioł et al., 2021; Santo et al., 2021; Wu et al., 2021). Although the effective composition of some extracts is still to be determined, fewer side effects of natural derivates compared with synthesized molecules due to their compatibility with humans should be noted. Certain natural products are promising in disease treatment as they also have an effect on autophagy, apoptosis, inflammation, or oxidative stress pathways (Zhang et al., 2020; Kesh et al., 2021b; Santo et al., 2021; Wu et al., 2021). Recently, Vaz *et al.* tested 1600 FDA-approved bioactive drugs in the 6-OHDA-induced zebrafish model and highlighted novel molecules with antiparkinsonian potential (Vaz et al., 2020). These drugs could possibly become adjuvant medicine in the future treatment of PD. New approaches such as nanoparticles for drug discovery are also being developed in zebrafish models. For example, a nanocrystal formulation of resveratrol protects DA neurons against cytotoxicity induced by MPP^+^ with no significant toxic effects on zebrafish embryos and larvae (Xiong et al., 2020).

## Amyotrophic Lateral Sclerosis

ALS is a neurodegenerative disease pathologically characterized by progressive loss of upper motor neurons from the cortex or lower motor neurons from the spinal cord and brainstem. The clinical features of ALS patients are first manifested by muscle weakness, hyperreflexia, fasiculation, atrophy, spasticity, and eventually resulting in paralysis. The pathophysiology mechanism of ALS may be due to excitotoxicity, the occurrence of astrogliosis and microgliosis, failure of proteostasis, mitochondrial dysfunction, neuroinflammation and so on (Van Damme et al., 2017). Familial ALS only accounts for 10% of all cases, while most ALS cases are sporadic. Genetic mutations and environmental factors are suggested to be the causes of ALS (Morrice et al., 2018a). Based on these findings, Tg and toxin-induced animal models of ALS have been established to investigate the disease pathogenesis or discover potential drugs.

### Neurotoxic Zebrafish Models of Amyotrophic Lateral Sclerosis

Environmental factors such as β-N-methylamino-l-alanine (BMAA) and bisphenol A (BPA) have been demonstrated to be involved in ALS etiology (Gois et al., 2020). BPA is an industrial plasticizer which is thought to be a potential trigger in ALS pathogenesis. It can cause motor neuron degeneration, affect locomotor activity, reduce neuromuscular junction (NMJ) integrity and motor neuron-specific cell death in zebrafish embryos (Morrice et al., 2018b). BMAA is a non-proteinogenic neurotoxic amino acid produced by cyanobacteria. Zebrafish show neurodegeneration symptoms when exposed to BMAA, including neural development disruption and learning and memory ability deficiency (Chiu et al., 2011; Wang et al., 2020b). The mixture of cyanotoxins, BMAA and microcystin leucine and arginine (MCLR), cause severe neurotoxicity and upregulation of TDP-43 in zebrafish than individual toxins (Martin et al., 2021). Together, this suggesting a link between cyanotoxins and ALS. Furthermore, the combined effects of ALS causative genes and environmental factors have also been investigated in ALS zebrafish models. Exposure to BPMM changes the motor neuron growth characteristics in zebrafish with G93R-SOD1 mutation (Sher, 2017). However, neurotoxin-induced ALS-like models are used less than Tg models in drug discovery.

### Genetic Zebrafish Models of Amyotrophic Lateral Sclerosis

Mutations on the genes, *SOD1* (Cu/Zn superoxide dismutase 1), *TARDBP* (TAR DNA-binding protein 43), *FUS/TLS* (fused in sarcoma/translocated in liposarcoma), and *C9ORF72* have been identified in ALS patients. SOD1 is an enzyme which catalyzes the detoxification of superoxide. Mutation SOD1-G93A impacts zebrafish motor neuron outgrowth, axonal branching, NMJ integrity, motor neuron survival and motor activity. SOD1-G93R mutation shows slowly progressive motor degeneration without muscle denervation in zebrafish (Sakowski et al., 2012; Morrice et al., 2018a). Good representation of ALS phenotype in zebrafish carrying these two mutations suggests a latent model for further drug screening. TDP43 is a nuclear protein which is associated with protein aggregation of motor neurons in ALS patients and is involved in axonal transport. *TARDBP-A315T* mutation in zebrafish shows motor dysfunction and motor axon abnormality (Morrice et al., 2018a). Knockdown of TDP43 ortholog tdp-43 in zebrafish not only causes early defects of motor phenotype with NMJ disassembly, but also decreases AChE expression in total fish (Campanari et al., 2021). Overexpression of tdp-43 in spinal motor neurons halts axonal outgrowth coupled with protein TDP-43 cytoplasmic mis-localization instead of aggregation (Asakawa et al., 2020). *FUS/TLS* gene encodes sarcoma fusion protein, which is responsible for RNA-binding. A truncated mutation of FUS-R495X in zebrafish leads to cytoplasmic protein accumulation in motor neurons as well as oxidative stress (Bosco et al., 2010). Depletion of FUS ortholog in zebrafish models results in the main characteristics of ALS physiopathology, including impaired motor abilities, shortened motor neuron length and NMJ fragmentation (Bourefis et al., 2020). The *C9ORF72* gene possesses intronic hexanucleotide-repeat (GGGGCC) expansions, which can be translated into aggregation-prone DPR (dipeptide-repeat) proteins, thus leading to neuronal toxicity (Kramer et al., 2018). Shaw *et al.* generated two zebrafish lines expressing C9orf72 hexanucleotide-repeat expansions and validated their disease-like symptoms of ALS (Shaw et al., 2018). Zebrafish from another group stably expressing C9orf72-associated 100 Gly-Arg repeats in motor neurons also displayed a reduction in motor neuron length and swimming capability (Swaminathan et al., 2018).

### Drug Discovery in Zebrafish Models of Amyotrophic Lateral Sclerosis

Zebrafish models also accelerate the development of new drug candidates for the treatment of ALS (Table 3).

**TABLE 3:** Drugs identified for ALS treatment in zebrafish models.

Compound	Classification	Model	Effects on phenotype	Behavioral method	Molecular mechanism	References
FPL64176 and Bay K8644	L-type calcium channel agonists	TARDBP-G348C-Tg	Rescue impaired locomotor function	Touch-evoked escape response, 52–56 hpf	Rescue motoneuron excitability, decrease synaptic fidelity, NMJ abnormal structures	Armstrong and Drapeau, (2013)
Pimozide	T-type Ca^2+^ channels antagonist	TARDBP-G348C-Tg; SOD1-G93A-Tg; FUS-R521H-Tg	Improve motor defects, stabilize neuromuscular transmission	Touch-evoked escape response, 52–56 hpf	Not applicable	Patten et al. (2017)
TRVA242	Pimozide derivatives	TARDBP-G348C-Tg; SOD1- G93A-Tg; C9ORF72	Restores aberrant spinal motor neuron outgrowth; recovers NMJ structures and synaptic deficits	Locomotion test, 5 dpf	Not applicable	Bose et al. (2019)
Riluzole	Sodium current I_NaP_ inhibitor	SOD1-G93R-Tg	Reverse abnormal movements	Touch-evoked escape response, 96 hpf; locomotion test, 12 months	Reduce neuronal stress	Benedetti et al. (2016)
Methylene blue	Cationic thiazine dye	TARDBP-G348C-Tg and FUS-R521H-Tg	Protect impaired motor phenotypes	Touch-evoked escape response, 48 hpf	Inhibit TDP-43 aggregates, suppress oxidative stress	Vaccaro et al. (2012)
1-Fe	Corrole iron complex	SOD1-G93R-Tg	Improve motor defects	Light-dark test, 6 dpf	Anti-antioxidant	Soll et al. (2021)
Ciprofloxacin and celecoxib	Antibiotics and anti-inflammatory	SOD1-G93R-Tg; TARDBP-G348C-Tg	Rescue motor defects and axonopathy, recover NMJ structures and ramified morphology of microglia	Touch-evoked escape response, 54–56 hpf; light-dark test, 6 dpf	Anti-inflammatory	Goldshtein et al. (2020)
Terbutaline sulfate	β2-adrenergic receptor agonist	TARDBP-Q331K-Tg	Prevent axonal defects and NMJ degeneration	Not applicable	Activate β2-adrenergic receptors	Paik et al. (2015)
PFI-1	Bromodomain inhibitor	PR_20_	Improve embryos viability	Not applicable	Not applicable	Corman et al. (2019)
Na-Phen	Histone deacetylase inhibitor	PR_20_	Improve embryos viability	Not applicable	Not applicable	Corman et al. (2019)
Telbivudine	Uracil-like nucleoside compound	SOD1-W32S-Tg	Rescue axonopathy and motor neuron deficits	Touch-evoked escape response, 48 hpf	Interact with W32 residue	DuVal et al. (2019)

Modulation of ion channels or currents sheds light on ALS treatment and drug discovery. L-type calcium channel agonists, FPL64176 or Bay K8644 to treat ALS have been evaluated in TARDBP Tg zebrafish with G348C mutation. Mutant TARDBP zebrafish showed impaired locomotor function, increased motoneuron excitability, decreased fidelity of synaptic transmission, and abnormal structures at NMJ. Calcium channel agonists treatment of zebrafish can rescue all these behavioral and cellular deficiencies (Armstrong and Drapeau, 2013). The neuroleptic compound, pimozide functions to antagonize T-type Ca^2+^ channels. Pimozide not only reduces motor defects, rescues orphaned presynaptic endings and stabilizes neuromuscular transmission in zebrafish expressing mutant TDP-43, but also reduces motor deficits in human SOD1-G93A and FUS-R521H Tg zebrafish (Patten et al., 2017). A correlative study screened out one potent compound TRVA242 from a pool of pimozide derivatives. TRVA242 prevented locomotor defects in four Tg zebrafish models, a human TARDBP-G348C mutant model, human SOD1-G93A mutant model, zebrafish *tardbp* loss-of-function model and a C9ORF72 model with 100 times Gly-Arg repeat. Moreover, TRVA242 restores aberrant spinal motor neuron outgrowth, recovers NMJ structural abnormalities, and rescues NMJ synaptic deficits (Bose et al., 2019). Notably, riluzole, which can inhibit persistent sodium current I_NaP_, reverses the abnormal motor phenotype, normalizes motor axon length, reduces the frequency of spontaneous tail coiling and depolarization frequency of spinal neurons in zebrafish embryos with SOD1-G93R mutation (Benedetti et al., 2016). Another study corroborated the mutant SOD1-G93R zebrafish as a valid ALS animal model for high-throughput drug screening and provided evidence for riluzole in reducing neuronal stress (McGown et al., 2016).

Mechanisms of ALS prevention are complicated and not as distinct as those for AD or PD. Oxidative stress, inflammatory, autophagy or other pathway interference seems effective in alleviating ALS symptoms. In identifying other potential neuroprotective compounds, methylene blue, which has been previously demonstrated to inhibit TDP-43 aggregates formation, was found to protect against oxidative stress and impaired motor phenotypes in mutant human TARDBP-G348C or FUS-R521H zebrafish models (Vaccaro et al., 2012). 1-Fe, acting as an corrole iron complex and catalytic antioxidant, also shows improved locomotor activity in SOD1-G93R mutant zebrafish (Soll et al., 2021). Interestingly, in a recent study, two ALS Tg zebrafish models, SOD1-G93R and TARDBP-G348C, were used to evaluate the combined efficacy of ciprofloxacin and celecoxib. Ciprofloxacin is an approved synthetic antibiotic and celecoxib is an approved nonsteroidal anti-inflammatory drug. Combination treatment improved zebrafish larvae performance and motor neuron axonopathy, and recovered the ramified morphology of microglia and NMJ structures (Goldshtein et al., 2020). These results provide a novel view of drug combination as an effective and promising treatment for ALS. Paik *et al.* predicted terbutaline sulfate (TS), widely used for asthma, to be a promising candidate for ALS by analyzing electronic medical records and genomics data. TS can prevent axonal defects and NMJ degeneration in a dose-dependent manner in TARDBP-Q331K-Tg zebrafish. The therapeutic effects of TS are mediated by activating β2-adrenergic receptors (Paik et al., 2015). Mutation of *SQSTM1* has been recently reported in ALS patients. *SQSTM1* knockdown in zebrafish causes locomotor deficits and axonopathy of motor neurons. However, treatment with rapamycin, an mTOR-dependent autophagy activator, rescues motor deficiency (Lattante et al., 2015). A method based on reducing C9ORF72 DPR toxicity is used for drug screening. The ALS-related polypeptide, PR_20_ is a synthetic polymer of DPR and has been demonstrated to be toxic in K562 cells and mouse primary neurons (Kramer et al., 2018). PFI-1 BET (bromo and extra-terminal) is a bromodomain inhibitor and Na-Phen is a histone deacetylase inhibitor. Both of these inhibitors can increase the viability of zebrafish embryos *in vivo* when exposed to PR_20_ (Corman et al., 2019). A tryptophan residue at position 32 (W32) in SOD1 is suggested to contribute to SOD1 induced toxicity in ALS. Zebrafish embryo with an uncommon SOD1-W32S variant shows axonopathy and motor neuron deficits. A uracil-like nucleoside compound, telbivudine, was predicted to interact with W32 residue. Telbivudine can rescue the phenotypes induced by SOD1-WT or disease-associated mutant in a dose-dependent manner (DuVal et al., 2019).

## Huntington’s Disease and Prion-Related Diseases

Huntington’s disease (HD) is a monogenic neurodegenerative disease caused by the mutation encoding for an abnormal trinucleotide that leads to glutamine (CAG) expansion at the Huntingtin (HTT) protein (Walker, 2007). Deficient HTT expression in zebrafish results in a variety of developmental defects and disruption in iron homeostasis (Lumsden et al., 2007). Zebrafish embryos expressing the expanded polyglutamine repeat versions cause various pathologies including early cell death and neurodegeneration. Overexpression of the C-terminal Hsp70-interacting protein (CHIP) can suppress aggregation and toxicity of pathogenic HTT fragment (Miller et al., 2005). The zebrafish HD model has also been used to screen for the molecular chaperones with anti-prion aggregation and toxicity effects (Schiffer et al., 2007). Furthermore, recent study revealed that the inhibitors of phosphodiesterase 5 (PDE5), which upregulated intracellular levels of cGMP, reduced aggregates in zebrafish larvae expressing human huntingtin with 71 glutamine repeats (VerPlank et al., 2020). We expect that the zebrafish model of HD will substantially improve our understanding of the disease and promote the development of novel drug discovery in HD.

Prion diseases are characterized by the aggregation of a pathogenic misfolded form of prion protein (PrP) which has a gain of toxic function. Moreover, accumulating evidence show that PrP may act as a receptor for protein aggregates and play a key role in the pathogenesis of other common neurodegenerative disorders (Watts et al., 2018). Using genetic zebrafish models, researchers have found that APP may control neuronal excitability via their interaction with the prion protein (Kanyo et al., 2020). Longer Aβ oligomers or protofibrils can also directly bind to PrP and thereby act to modulate synapses (Um et al., 2012). Blockade of the binding can prevent sleep induction in zebrafish, resulting in a signaling cascade to exacerbate AD progression (Özcan et al., 2020). Taking together, PrP protein could be a potential pharmaceutical target for the treatment of prion diseases and other neurodegenerative disorders.

## Conclusion and Remarks

As the number of people affected by NND is increasing, finding an effective way of treating the disease is urgently needed. However, only symptomatic treatment is available to date. Current AD therapies can only temporarily improve learning and memory by maintaining the levels of ACh and cholinergic transmission by AChE inhibitors or regulating glutamatergic transmission by blockers of N-methyl-d-aspartate (NMDA) receptors. Administration of l-DOPA, dopamine agonists, MAO inhibitors, catechol-O-methyltransferase (COMT) inhibitors and anticholinergics has been shown to temporarily relieve motor and nonmotor PD symptoms but without halting PD progression. In ALS, FDA-approved drugs (riluzole and edaravone) only provide a modest survival benefit. A number of pharmacological drugs designed for NDD treatment have failed in clinical trials over the past several decades due to the clinical heterogeneity of the disease. In addition, disease-modifying treatments can only be effective in the early stage of the disease because most NDD pathologies are irreversible. Therefore, we need to generate more pre-clinical NDD animal models to mimic the alteration in different clinical subtypes and in different stages of the disease. To accomplish this, the zebrafish model is essential. Zebrafish have a number of advantages and provide an important foundation for the determination of NDD pathogenesis and drug discovery. *In vitro* chemical screening provides much higher throughput than *in vivo*, while *in vivo* models can simulate complex systems in the human body such as the BBB. Zebrafish have been used in both *in vitro* and *in vivo* drug screening, and numerous new compounds have been verified to be effective against NDD.

However, there is still the challenge of translating these drugs into clinical use. First, there is a big gap between zebrafish and humans in terms of NDD morbidity and drug treatment. Even in Tg zebrafish models, the pathological or behavioral phenotypes are varied due to different genes or mutations. Second, the pathogenesis of NDD involves a host of environmental and genetic factors; thus, classifying the disease into different subtypes. It may be necessary to personalize the treatment approach. Third, the lack of sensitive biomarkers limits the identification of NDD at pre-symptomatic stages, in which drug treatment is likely to show disease-modifying efficacy. In addition, many drugs screened out in zebrafish models have a short therapeutic window. It is difficult for these drugs to access the BBB before degradation without suitable drug delivery approaches.

Small molecules from natural extracts or designed targeting a critical pathway are both feasible for NDD treatment. Although many compounds show efficiency at different levels in alleviating zebrafish pathological or aberrant behavioral features in NDD, comprehensive testing should be carried out to fully understand the mechanisms of a new drug. It is unknown how many NDD abnormal symptoms these drugs have an effect on. This would help us to predict the viability of drugs for further trials. In addition, more molecular mechanisms should be identified in order to develop or discover new drugs besides existing inhibitors or targeting proteins in NDD. Drugs with little or no specificity should also be investigated and improved. Lastly, pharmacological drug safety of compounds should be determined. Zebrafish will be helpful in the transfer of drugs from the laboratory to the clinic.

## Glossary

6-OHDA: 6-hydroxydopamine
α-syn: α-synuclein
AChE: acetylcholinesterase
AD: alzheimer’s disease
ALS: amyotrophic lateral sclerosis
AlCl3: aluminum trichloride
Aβ: β-amyloid
APP: amyloid precursor protein
ASOs: antisense oligonucleotides
ATG5: autophagy-related gene 5
BACE1: β-secretase 1
BBB: blood-brain barrier
BET: bromo and extra-terminal
BSP: bromosporine
BuChE: butyrylcholinesterase
CaP-lipid NPs: calcium phosphate lipid coated nanoparticles
CDK5: cyclin-dependent kinase 5
CQDs: carbon quantum dots
DA: dopamine
FA: 4-hydroxy-3-methoxycinnamic acid
FUS: fused in sarcoma
Hpf: hours post fertilization
IAPP: islet amyloid polypeptide
GSI: γ-secretase inhibitor
GSK 3β: kinase glycogen synthase 3β
LBs: Lewy bodies
L-BMAA: beta-N-methylamino-l-alanine
LKE: lanthionine ketimine-5-ethyl ester
LRRK2: leucine-rich repeat kinase 2
MAO: monoamine oxidase
MAPT: microtubule-associated protein tau
MCU: mitochondrial calcium uniporter
MPP+: 1-methyl-4-phenylpyridinium ion
MPTP: 1-methyl-4-phenyl-1,2,3,6-tetrahydropyridine
Na-Phen: sodium phenylbutyrate
NDD: neurodegenerative disease
NFTs: neurofibrillary tangles
NMJ: neuromuscular junction
OKA: okadaic acid
PD: Parkinson’s disease
SNpc: substantia nigra pars compacta
SOD1: superoxide dismutase 1
ROS: reactive oxygen species
TARDBP: TAR DNA-binding protein 43
TDZD-8: 4-benzyl-2-methyl-1, 2, 4-thiadiazolidine-3, 5-dione
Tg: transgenic model
TH: tyrosine hydroxylase
TLS: translocated in liposarcoma
